# Relationship of the metabolic score for insulin resistance and the new-onset hypertension: Evidence from CHARLS

**DOI:** 10.1371/journal.pone.0336388

**Published:** 2025-11-07

**Authors:** Chun-Fang Ma, Xiang-Xiang Li, Shan Liu, Xiao-Fei Wu

**Affiliations:** 1 Department of Clinical Laboratory, Suzhou Ninth Hospital affiliated to Soochow University, Suzhou, Jiangsu, China; 2 Department of Emergency Medicine, Suzhou Ninth Hospital affiliated to Soochow University, Suzhou, Jiangsu, China; Tehran University of Medical Sciences, IRAN, ISLAMIC REPUBLIC OF

## Abstract

**Background:**

Hypertension (HTN) progression is linked to insulin resistance (IR), yet the association between Metabolic Score for Insulin Resistance (METS-IR) and HTN remains underexplored.

**Methods:**

This study included 4,051 individuals without a history of HTN from the China Health and Retirement Longitudinal Study (CHARLS). Participants were stratified into four groups according to their baseline METS-IR values. It was the development of incident HTN that was the primary outcome. We used Cox regression to assess this association, conducted subgroup and sensitivity analyses, and evaluated METS-IR’s incremental predictive value over conventional risk factors (age, sex, systolic blood pressure) using C-statistic, net reclassification improvement (NRI), integrated discrimination improvement (IDI), and decision curve analysis (DCA).

**Results:**

Over the 9-year follow-up, 1,572 participants (38.81%) experienced their first incident of HTN. Participants were categorized into quartiles (Q1-Q4) based on their METS-IR levels. After full adjustment for confounders, the hazard ratio (HR) with a 95% confidence interval (CI) for incident HTN demonstrated a progressive increase across ascending METS-IR quartiles, with Q1 as reference: Q2, 0.99 (0.85–1.15); Q3, 1.17 (1.01–1.36); Q4, 1.31 (1.13–1.52). The restricted cubic spline (RCS) model revealed a linear dose-response relationship between METS-IR and the incidence of HTN (*P* for overall trend < 0.001; *P* for nonlinear = 0.310). Adding METS-IR to a base model (age/sex/systolic blood pressure) improved HTN prediction (C-statistic Δ= + 0.004; NRI = 16.58%, IDI = 0.75%; all *P* < 0.001). DCA confirmed greater net benefit across risk thresholds. Subgroup and sensitivity analyses consistently supported the primary findings.

**Conclusion:**

Elevated METS-IR independently predicts HTN risk in Chinese adults, suggesting METS-IR as a potential indicator.

## Introduction

Hypertension (HTN) is a major cardiovascular risk factor, increasing adverse events, cardiac mortality, stroke, and chronic kidney disease risk [1]. Affecting ~244 million adults in China, HTN poses a major public health challenge despite management efforts [2]. Early identification of high-risk individuals requires understanding key risk factors, including obesity, dyslipidemia, and insulin resistance (IR) [3].

IR is a key pathophysiological mechanism underlying HTN and its frequent comorbidity, type 2 diabetes mellitus (T2DM) [4–6]. While the hyperinsulinemic-euglycemic clamp (HEC) is the gold standard for assessing insulin sensitivity, its complexity limits widespread use [7]. Alternative indices like HOMA-IR still require insulin measurement, hindering routine screening [8]. This spurred development of non-insulin-dependent IR indices using readily available data.

The Metabolic Score for Insulin Resistance (METS-IR) is a novel, non-insulin-based metric integrating fasting plasma glucose (FPG), triglycerides (TG), high density lipoprotein cholesterol (HDL-C), and body mass index (BMI) [9]. It offers a comprehensive reflection of obesity-driven metabolic dysfunction—a core mechanism in HTN and other non-communicable diseases (NCDs) like cardiovascular disease (CVD), fatty liver, and metabolic syndrome. METS-IR specifically aids in identifying early metabolic dysregulation pathways central to NCDs development, positioning it as a valuable tool for NCDs risk stratification and prevention [9–14]. Compared to other non-insulin indices—such as the TyG index (TG and glucose-focused) and TG/HDL-C ratio (lipid-centric)—METS-IR’s inclusion of BMI better captures the critical interplay between adiposity and metabolic dysregulation central to NCDs pathogenesis [15,16]. However, prospective evidence for METS-IR’s ability to predict incident HTN in Chinese middle-aged/elderly adults (≥45 years) remains scarce. This population exhibits unique metabolic traits (e.g., higher visceral adiposity at lower BMIs, salt sensitivity) [17,18], and prior evidence is largely limited to cross-sectional studies [4,19,20].

To address this gap, we leveraged the nationally representative China Health and Retirement Longitudinal Study (CHARLS) cohort to determine whether METS-IR independently predicts HTN development. We hypothesized elevated baseline METS-IR would show a linear, positive association with incident HTN risk in Chinese adults, independent of traditional cardiovascular risk factors, and evaluated this using CHARLS prospective data.

## Materials and methods

### Data source

Participants were recruited from the CHARLS study, a nationwide prospective cohort study that focuses on residents aged 45 years and older in both urban and rural areas of China [21]. This research was carried out in accordance with the principles outlined in the Declaration of Helsinki, and received approval from the Ethics Committee of Peking University (IRB00001052–11015). Approval for data collection was obtained from the Ethical Review Committee at Peking University. The participants provided their written informed consent to participate in this study. This secondary analysis of de-identified CHARLS records required neither renewed consent nor ethics committee approval, as it involved no patient privacy risks. The current study’s dataset is available for public access on the following website: http://charls.pku.edu.cn/en.

### Study participants

**Fig 1:**
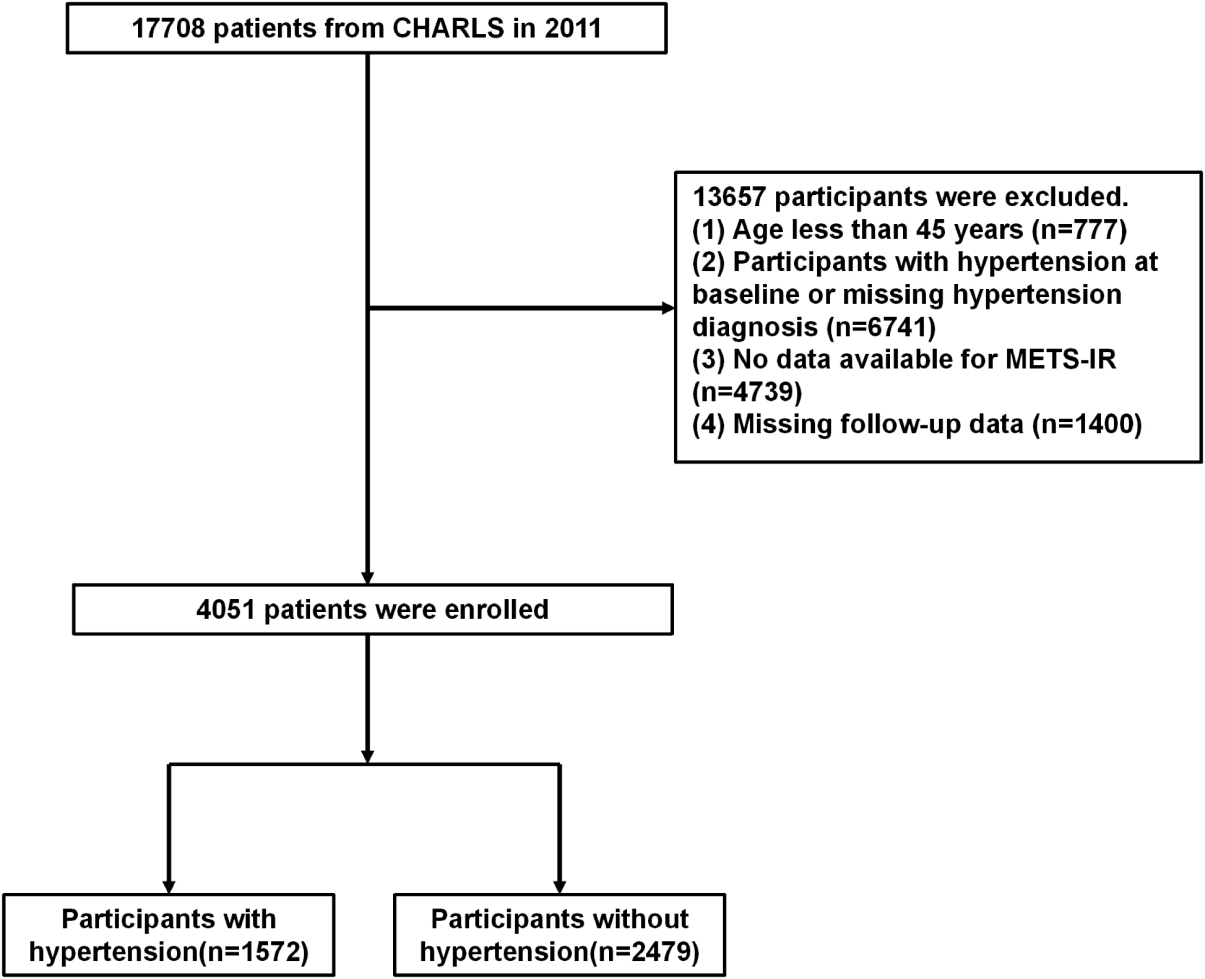
Flowchart of the study population.

This study involves a biennial survey, with data collected in 2011, 2013, 2015, 2018, and 2020. The current research integrates data from all five survey iterations. Among the 17,708 participants, 4,051 were selected based on eligibility criteria and subsequently categorized into four quartile groups based on their METS-IR values. Exclusion criteria encompassed: (1) age < 45 years (n = 777); (2) pre-existing HTN or missing HTN diagnosis at baseline (n = 6,741); (3) missing data required for METS-IR calculation (n = 4,739); and (4) missing follow-up data (n = 1,400), resulting in a final analytical cohort of 4,051 participants (hypertensive: n = 1,572; normotensive: n = 2,479), with complete selection procedures detailed in **Fig 1**.

### Covariates ascertainment

Trained interviewers collected data on core domains—including demographics, family structure, health status, chronic conditions, lifestyle, and health behaviors—through standardized questionnaires administered per established protocols. Comprehensive methodological details are available in the published cohort profile [22]. Demographic variables encompassed age, gender, residence (rural/urban), marital status (married or living with spouse/non-married), along with anthropometric parameters including BMI (kg/m²; calculated as weight/height²). Behavioral features incorporated smoking status (classified as “No” for never smokers vs. “Yes” for former/current smokers [23]) and alcohol consumption (categorized as “No” or “Yes” based on past-year intake [24]). Health status documentation covered diabetes mellitus (DM) [25], dyslipidemia [26], and heart disease [27]. The criteria for the diagnosis of DM encompass FPG levels of 126 mg/dL or higher, HbA1c values of 6.5% or greater, a clinical diagnosis made by a healthcare professional, or the prescription of antidiabetic medications. The diagnostic criteria for dyslipidemia encompass a self-reported diagnosis by a physician, the current use of lipid-lowering medications, and the following laboratory values: total cholesterol (TC) ≥ 240 mg/dL, TG ≥ 150 mg/dL, HDL-C < 40 mg/dL, and low density lipoprotein cholesterol (LDL-C) ≥ 160 mg/dL. The enzymatic colorimetric assay was employed to determine FPG levels and serum lipid parameters. Glycated hemoglobin (HbA1c) was quantified using boronate affinity high-performance liquid chromatography.

### Outcome ascertainment

Participants were instructed to rest in a seated position for at least 5 minutes before the assessment. Blood pressure (BP) was measured by trained interviewers using a calibrated Omron HEM-7200 digital sphygmomanometer after ≥5 minutes of seated rest. Three measurements were taken at 45-second intervals, and the average of these readings was used for analysis [22]. Field staff followed a standardized protocol; blinding to metabolic predictors was not applicable. BP measurements were obtained during the 2011, 2013, and 2015 surveys: a trained interviewer recorded three BP measurements at 45-second intervals using a digital sphygmomanometer. For the 2018 and 2020 surveys, BP was not measured; thus, HTN status was assessed solely by self-reported diagnosis and medication use.

HTN is diagnosed by meeting at least one of the following criteria [28]: self-reported physician diagnosis; current use of antihypertensive medications; or measured systolic blood pressure (SBP) ≥140 mmHg and/or diastolic blood pressure (DBP) ≥90 mmHg. In this statistical analysis, the time of initial HTN diagnosis was considered as the onset time. Incident HTN was defined as the first occurrence during follow-up of any diagnostic criterion. The event date was assigned as the exact visit date when HTN was first identified, consistent with: a) The practical limitation that exact onset timing between visits is unobservable, and b) Standard cohort methodology where detection time defines actionable disease status.

### Calculation of METS-IR

The arithmetic equation for METS-IR is expressed as follows, METS-IR components (FPG, TG, HDL-C) were measured in mg/dL. Natural logarithms (Ln) were applied to normalize distributions and align with the original METS-IR derivation [9]:

METS-IR = Ln [2 × FPG (mg/dL) + TG (mg/dL)] × BMI (kg/m²)/ Ln [HDL-C (mg/dL)] (1)

### Missing data handling

This study ensured analytical rigor through a multi-faceted validation framework: Missing data management entailed the exclusion of participants with incomplete METS-IR components; complete-case analysis was retained as the primary approach due to METS-IR’s composite nature, with validation through Multiple Imputation by Chained Equations (MICE); Statistical power was validated by assessing minimum detectable effects and comparing them with observed effect sizes; Attrition bias due to loss to follow-up was addressed through baseline characteristic comparisons between retained and excluded participants combined with Inverse Probability of Censoring Weighting (IPCW) Cox regression applied to the full cohort using baseline covariates, effectively accounting for potential selection bias from participant attrition.

### Statistical analysis

This study adhered to the STROBE guidelines for reporting observational research [29]. Continuous variables were expressed as mean ± standard deviation (SD) or median (interquartile range), and categorical variables were reported as frequencies and percentages. Comparisons between groups for normally distributed, skewed, and categorical variables were performed using one-way ANOVA, the Kruskal-Wallis test, and the chi-square test, respectively. Participants were classified into four groups according to their METS-IR quartile levels: Quartile 1 (Q1), with METS-IR ≤ 29.03; Quartile 2 (Q2), with 29.03 < METS-IR ≤ 32.95; Quartile 3 (Q3), with 32.95 < METS-IR ≤ 38.15; and Quartile 4 (Q4), with METS-IR > 38.15. The cumulative incidence of incident HTN was assessed using Kaplan-Meier curves and log-rank tests. The association between METS-IR and incident HTN was assessed using multivariable Cox regression analysis. After considering potential confounding factors, we utilized four distinct models for analysis: the unadjusted model controlled for no variables; Model 1 adjusted for demographic and lifestyle factors (age, gender, marital status, residence type, smoking status, and alcohol consumption); Model 2 further incorporated clinical biomarkers and comorbidities (BUN, serum creatinine, TC, LDL-C, CRP, UA, dyslipidemia, heart disease, and DM) into Model 1; and Model 3 expanded upon Model 2 by additionally adjusting for SBP/DBP. Proportional hazards assumptions were assessed using Schoenfeld residuals. Furthermore, to verify the stability of the primary association and address potential violations of the proportional hazards assumption for the covariate ‘sex’, a stratified analysis by gender was conducted as a sensitivity measure. To flexibly model the dose-response relationship between METS-IR (continuous) and incident HTN, we employed restricted cubic splines (RCS) with 5 knots placed at predefined percentiles (5th, 27.5th, 50th, 72.5th, 95th) of the METS-IR distribution. RCS overcomes limitations of linearity assumptions by partitioning the exposure into segments connected by polynomial functions, constrained to be linear beyond boundary knots. This approach captures potential nonlinear associations while minimizing overfitting. Knot positions were determined using the Harrell method to ensure sufficient data density across METS-IR values. The functional form was tested using Wald statistics for overall trend (*P* for trend) and nonlinearity (*P* for nonlinearity). To ensure the robustness of our findings, we performed three supplementary sensitivity analyses. First, participants with a BMI of 24 kg/m² or higher were excluded. Second, individuals with DM were also excluded. Finally, participants who were both overweight and had DM were excluded as well. Subgroup analyses were conducted based on baseline characteristics including age, gender, BMI, marital status, region, alcohol consumption status, smoking status, dyslipidemia, DM, and heart disease to evaluate the consistency of the adverse impact of METS-IR on the incidence of HTN. Interaction effects were assessed using likelihood ratio tests (LRT) comparing models with and without METS-IR-by-subgroup interaction terms. It should be noted that these interaction tests are exploratory in nature, and their interpretation should consider the potential for type I error due to multiple comparisons. To quantify the incremental predictive value of METS-IR beyond conventional HTN risk factors, we assessed discrimination improvement via change in Harrell’s C-statistic, net reclassification improvement (NRI), and integrated discrimination improvement (IDI) when adding METS-IR to a base model (age, sex, SBP). Decision curve analysis (DCA) was performed to evaluate clinical utility across risk thresholds (1%–99%). Model calibration, which assesses the agreement between predicted probabilities and observed outcomes, was evaluated using the calibration slope. A calibration slope close to 1.0 indicates good calibration. This analysis was performed using the rms package in R with bootstrap resampling (B = 500). To ensure the study was adequately powered to detect a clinically meaningful effect, the minimum detectable hazard ratio was calculated using the Schoenfeld formula for Cox proportional hazards models, given 1,572 incident events, 80% power, and a two-tailed α of 0.05. Statistical analysis was conducted using R software, version 4.1.2. Statistical significance was determined using a two-tailed test, with *P*-values less than 0.05 indicating statistically significant results.

## Results

### Data integrity and methodological robustness

The analytical rigor was ensured through a multi-faceted validation framework. Proportional hazards assumptions were confirmed via Schoenfeld residuals (global *P* = 0.104; METS-IR-specific *P* = 0.078, S1 Table), indicating no violation for either the primary exposure or overall model. Missing METS-IR components led to exclusion of 4,739 participants (S1 Fig), with Little’s MCAR test confirming non-random missingness (*P* < 0.001). Complete-case analysis was retained as the primary approach due to METS-IR’s composite nature, validated by MICE sensitivity analysis (20 imputations) showing minimal HR variation: complete-case HR = 1.21 vs. MICE HR = 1.19 (difference: 1.7%), both *P* < 0.001 with overlapping CI, underscoring robustness (S2 Table). Under 1,572 events, α = 0.05, and 80% power, the minimum detectable HR is 1.146. This indicates the study was adequately powered to detect clinically relevant effects. To specifically address potential attrition bias, we conducted an Inverse Probability of Censoring Weighting (IPCW) analysis as a sensitivity analysis. This analysis was applied to the eligible baseline cohort (n = 5,451), defined as all participants who met the inclusion criteria (i.e., after applying pre-follow-up exclusions for age, pre-existing HTN, and missing METS-IR components) but before the exclusion of individuals lost to follow-up. Baseline characteristics of the retained vs. lost-to-follow-up participants from this cohort are compared in S3 Table. Weights were derived from covariates predictive of attrition (S4 Table). IPCW analyses confirmed minimal variation in METS-IR effect estimates and robust associations after full adjustment for confounders: while the primary analysis showed a per-SD METS-IR increase corresponded to an HR of 1.13 (95% CI:1.08–1.19, Table 2), IPCW yielded an HR of 1.08 (95% CI:1.02–1.15; *P *< 0.001); similarly, for Q4 vs. Q1 comparisons, primary analysis indicated an HR of 1.31 (95% CI:1.13–1.52, Table 2), whereas IPCW results demonstrated an HR of 1.25 (95% CI:1.06–1.46; *P* = 0.006, S4 Table). Collectively, these measures ensured >80% power to detect clinically meaningful effects while maintaining robustness, reinforcing confidence in the primary findings.

### Demographic characteristics

Baseline clinical and demographic characteristics of participants, stratified by METS-IR quartiles, are presented in **Table 1**. Participants in the highest METS-IR quartile were generally younger, had a higher proportion of females, were more likely to be married, and had lower proportions of rural residents, current smokers, and current drinkers. The incidence of diabetes, heart disease, dyslipidemia, and HTN markedly escalates among individuals within the higher quartiles of METS-IR. Furthermore, with increasing METS-IR levels, participants exhibited significant increases in BMI, SBP, DBP, FBG, TC, TG, LDL-C, HbA1c, and UA levels, while HDL-C levels significantly decreased.

**Table 1:** Baseline characteristics of the study population by quartiles of METS-IR.

Variables	METS-IR					*P* value
	Overall (n = 4051)	Quartile 1 (n = 1013)	Quartile 2 (n = 1012)	Quartile 3 (n = 1013)	Quartile 4 (n = 1013)	
Age, years	56.00 (50.00-62.00)	58.00 (53.00-65.00)	57.00 (51.00-62.00)	55.00 (49.00-61.00)	55.00 (49.00-60.00)	<0.001
Male, n (%)	1837 (45.35%)	515 (50.84%)	496 (49.01%)	426 (42.05%)	400 (39.49%)	<0.001
Rural residence, n (%)	2801 (69.14%)	789 (77.89%)	729 (72.04%)	677 (66.83%)	606 (59.82%)	<0.001
BMI (kg/m^2^)	22.61 (20.54-24.90)	19.43 (18.33-20.49)	21.76 (20.74-22.73)	23.75 (22.62-24.82)	26.56 (24.87-28.38)	<0.001
SBP, mmHg	117.00 (108.50-126.50)	115.00 (106.00-124.50)	116.00 (108.00-125.50)	117.00 (109.00-125.50)	121.00 (112.00-129.00)	<0.001
DBP, mmHg	70.00 (64.00-76.50)	68.00 (61.50-74.50)	69.00 (63.00-75.00)	70.00 (64.50-76.50)	73.00 (66.50-79.00)	<0.001
Married or living with spouse, n (%)	3715 (91.71%)	911 (89.93%)	915 (90.42%)	936 (92.40%)	953 (94.08%)	0.002
Drinking, n (%)	1354 (33.42%)	379 (37.41%)	368 (36.36%)	311 (30.70%)	296 (29.22%)	<0.001
Smoking, n (%)	1228 (30.31%)	381 (37.61%)	337 (33.30%)	281 (27.74%)	229 (22.61%)	<0.001
BUN, mg/dL	15.10 (12.52-18.12)	15.74 (12.97-18.85)	15.35 (12.63-18.72)	14.90 (12.44-17.65)	14.59 (12.32-17.11)	<0.001
FBG, mg/dL	100.98 (93.60-110.16)	97.20 (90.54-105.12)	99.54 (93.06-107.32)	101.16 (94.14-110.16)	106.56 (97.92-121.14)	<0.001
Creatinine, mg/dL	0.75 (0.64-0.87)	0.75 (0.64-0.86)	0.75 (0.63-0.87)	0.73 (0.64-0.86)	0.75 (0.64-0.87)	0.837
TC, mg/dL	188.66 (165.85-213.02)	190.21 (167.01-212.24)	184.02 (162.76-211.08)	187.89 (165.46-211.86)	191.75 (168.17-216.50)	0.001
TG, mg/dL	98.24 (71.68-141.60)	74.34 (57.53-93.81)	87.17 (66.38-115.05)	104.43 (78.76-143.37)	157.53 (110.62-229.21)	<0.001
HDL, mg/dL	51.03 (41.75-61.08)	63.02 (55.28-73.45)	54.51 (47.17-62.63)	48.33 (41.75-55.28)	38.66 (33.25-46.01)	<0.001
LDL, mg/dL	113.66 (93.17-135.70)	110.57 (91.24-130.67)	112.89 (92.69-135.41)	117.53 (97.04-138.40)	114.43 (90.85-138.02)	<0.001
CRP, mg/L	0.87 (0.49-1.77)	0.67 (0.40-1.41)	0.76 (0.46-1.51)	0.89 (0.52-1.72)	1.22 (0.68-2.34)	<0.001
HbA1c, %	5.10 (4.90-5.40)	5.10 (4.90-5.30)	5.10 (4.90-5.30)	5.10 (4.80-5.40)	5.20 (4.90-5.60)	<0.001
UA, mg/dL	4.14 (3.46-4.94)	4.02 (3.34-4.81)	4.07 (3.42-4.91)	4.13 (3.49-4.86)	4.35 (3.64-5.17)	<0.001
Diabetes mellitus, n (%)	152 (3.75%)	13 (1.28%)	21 (2.08%)	44 (4.34%)	74 (7.31%)	<0.001
Heart diseases, n (%)	314 (7.75%)	59 (5.82%)	64 (6.32%)	86 (8.49%)	105 (10.37%)	<0.001
Dyslipidemia, n (%)	231 (5.70%)	23 (2.27%)	45 (4.45%)	73 (7.21%)	90 (8.88%)	<0.001
Hypertension, n (%)	1572 (38.81%)	347 (34.25%)	350 (34.58%)	393 (38.80%)	482 (47.58%)	<0.001

Data are means ± SD, median (interquartile range), or n (%). Pearson chi-square test or Kruskal–Wallis test or analysis of variance in multiple groups.

Abbreviations: METS-IR: metabolic score for insulin resistance, BMI: body mass index, BUN: blood urea nitrogen, SBP: systolic blood pressure, DBP: diastolic blood pressure, FBG: fasting blood glucose, HDL-C: high density lipoprotein cholesterol, LDL-C: low density lipoprotein cholesterol, TC: total cholesterol, TG triglycerides, CRP: C-reactive protein, HbA1c: glycosylated hemoglobin A1c, UA: uric acid.

### Predictive value of METS-IR in the onset of HTN

**Fig 2:**
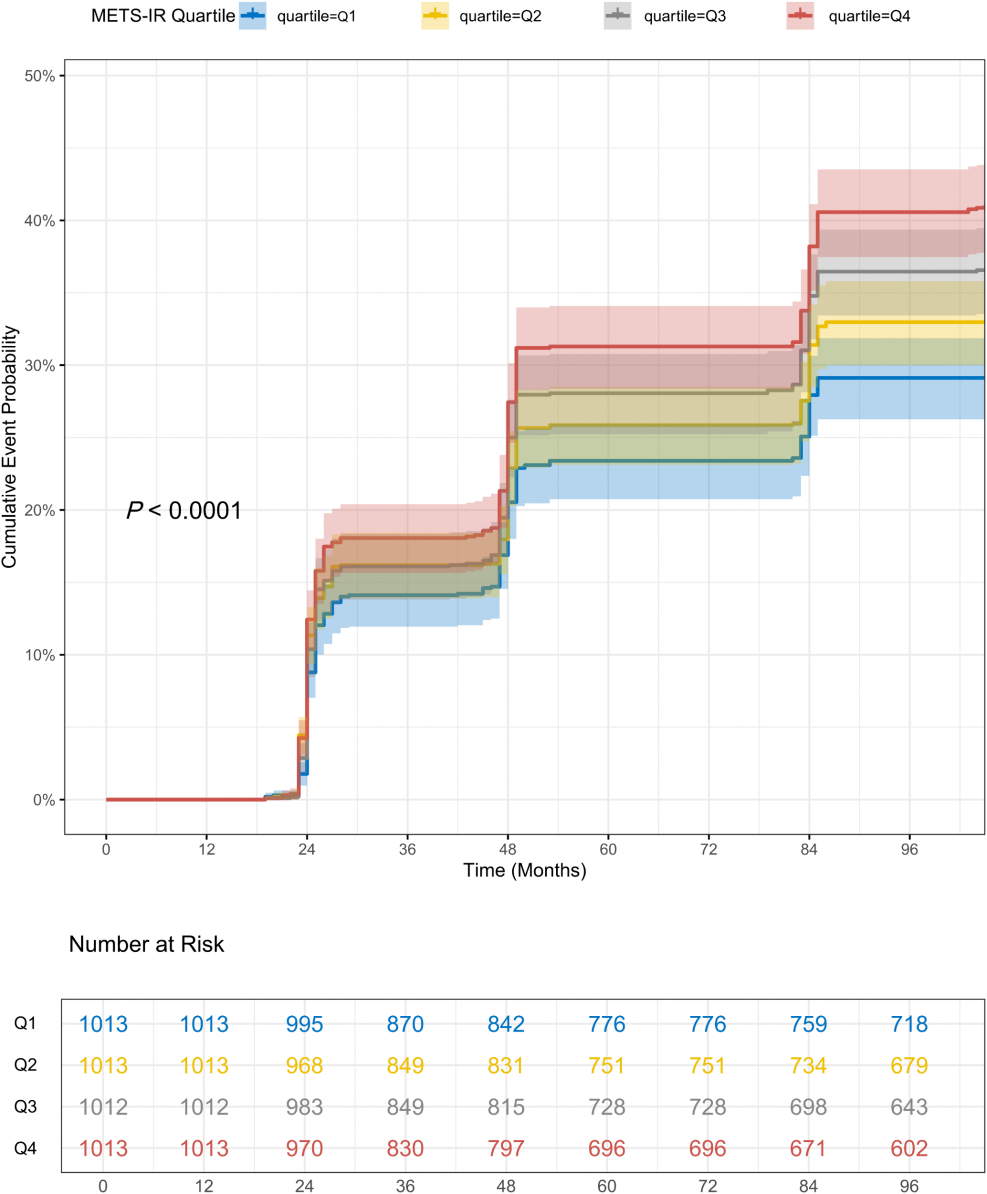
The Kaplan-Meier cumulative incidence curves for METS-IR and new onset hypertension.

**Fig 3:**
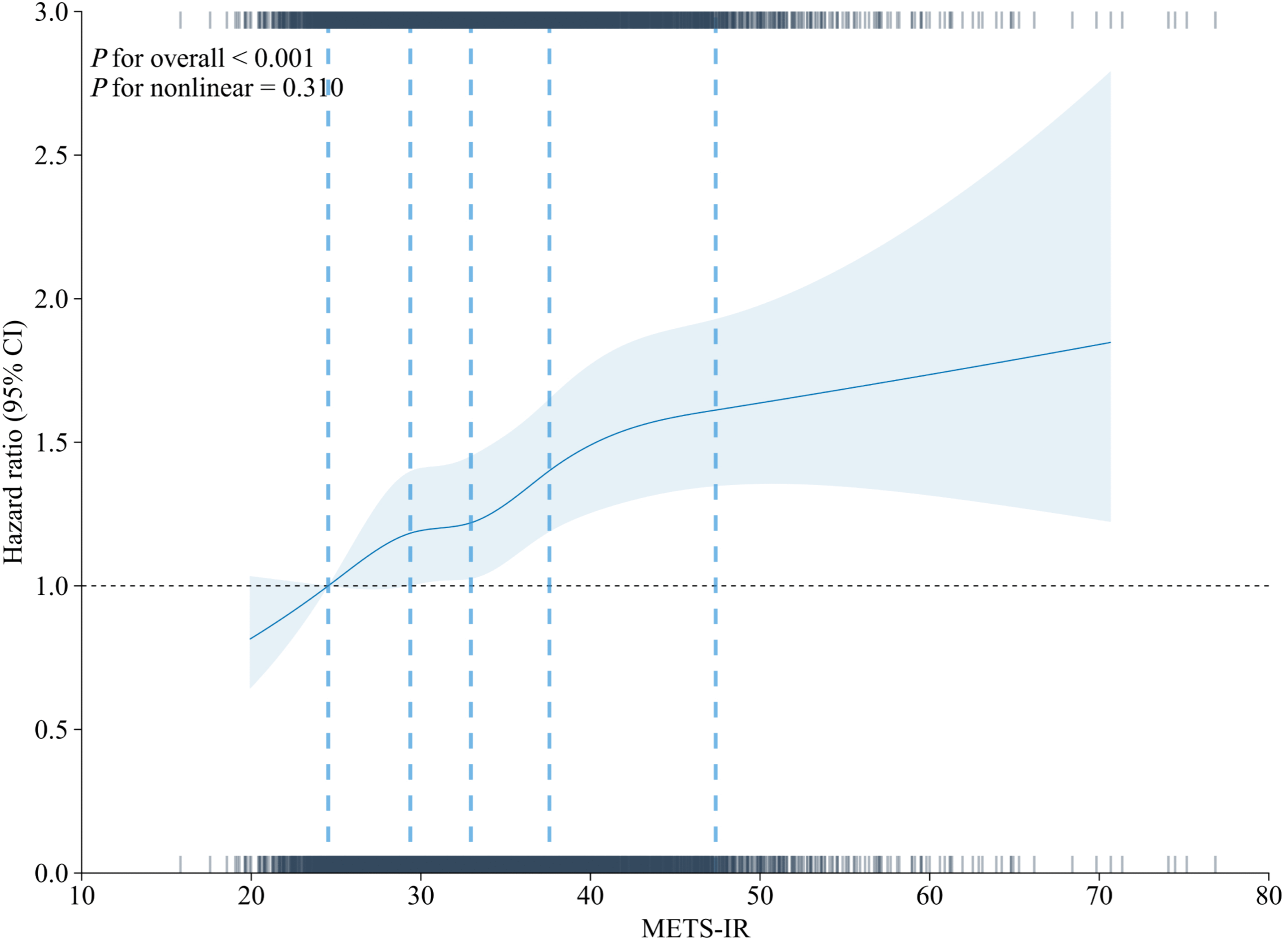
Analysis of the relationship between METS-IR values and hypertension risk using RCS methodology after covariate adjustment. Adjusted for age, gender, marital status, rural residence, smoking status and drinking status, BUN, serum creatinine, TC, LDL-C, CRP, UA, dyslipidemia, heart disease, diabetes mellitus, SBP and DBP. The linear trend was significant (*P* < 0.001) with no evidence of nonlinearity (*P* = 0.310). RCS: restricted cubic spline.

Over a nine-year follow-up period, 1,572 (38.81%) of these participants developed HTN for the first time. The overall HTN incidence rate was 43.12 per 1,000 person-years (95% CI: 40.99–45.25). The Kaplan-Meier cumulative incidence curves reveal a progressive increase in the incidence of HTN events from Group Q1 to Group Q4 (**Fig 2**, log-rank test *P* < 0.001). The Cox regression analysis presented in **Table 2** substantiates a significant association between baseline METS-IR and the incidence of new-onset HTN. Baseline METS-IR was evaluated both as a continuous measure and categorized into quartiles for analysis. After adjusting for potential confounders (model 3), Furthermore, compared to the lowest quartile (Q1) of METS-IR, the risk of HTN exhibited a progressive increase across higher quartiles: Q2 showed no significant association (HR = 0.99, 95% CI: 0.85–1.15; *P* = 0.887), Q3 demonstrated a 17% elevated risk (HR = 1.17, 95% CI: 1.01–1.36; *P* = 0.038), and Q4 had a 31% higher risk (HR = 1.31, 95% CI: 1.13–1.52; *P* < 0.001), with a statistically significant dose-response trend across quartiles (*P* for trend < 0.001; **Table 2**). The RCS analysis presented in **Fig 3** demonstrates a significant dose-response relationship between METS-IR as a continuous variable and the risk of HTN, after adjusting for multiple covariates (*P* for trend < 0.001; *P* for nonlinearity = 0.310).

**Table 2:** Cox models analyzed the relationship between METS-IR and the risk of hypertension.

	Non-adjusted model		Model 1		Model 2		Model 3	
	HR (95% CI)	*P* value	HR (95% CI)	*P* value	HR (95% CI)	*P* value	HR (95% CI)	*P* value
Continues								
Per SD increase	1.18 (1.13, 1.23)	<0.001	1.23 (1.18, 1.29)	<0.001	1.21 (1.15, 1.27)	<0.001	1.13 (1.08, 1.19)	<0.001
METS-IR quartile								
Q1	Ref		Ref		Ref		Ref	
Q2	0.99 (0.86, 1.15)	0.929	1.06 (0.92, 1.24)	0.408	1.06 (0.91, 1.23)	0.432	0.99 (0.85, 1.15)	0.887
Q3	1.14 (0.99, 1.32)	0.072	1.31 (1.13, 1.51)	<0.001	1.27 (1.10, 1.48)	0.001	1.17 (1.01, 1.36)	0.038
Q4	1.50 (1.31, 1.73)	<0.001	1.77 (1.54, 2.04)	<0.001	1.63 (1.41, 1.89)	<0.001	1.31 (1.13, 1.52)	<0.001
*P* for trend		<0.001		<0.001		<0.001		<0.001

HR: hazard ratios, CI: confidence interval, Ref: reference, METS-IR: metabolic score for insulin resistance.

Non-adjusted model adjusted for none.

Model 1 adjusted for age, gender, marital status, rural residence, smoking status and drinking status.

Model 2 adjusted for BUN, serum creatinine, TC, LDL-C, CRP, UA, dyslipidemia, heart disease and diabetes mellitus on the basis of Model 1.

Model 3 adjusted for SBP and DBP on the basis of Model 2.

### Incremental predictive value of METS-IR

Incorporating METS-IR into the base model (age, sex, SBP) yielded statistically significant but modest discrimination improvement: C-statistic increased from 0.676 (95% CI: 0.662–0.689) to 0.680 (Δ = 0.004, *P* < 0.001), with NRI = 0.1658 (95% CI: 0.1033–0.2283, *P* < 0.001) and IDI = 0.0075 (95% CI: 0.0047–0.0103, *P* < 0.001, **Table 3**). Furthermore, both models demonstrated excellent calibration. The calibration slope was 0.987 for the base model and 0.992 for the base model plus METS-IR, indicating that the predicted risks were highly consistent with the observed outcomes. Incorporating METS-IR into the base model (age, sex, SBP) enhanced clinical decision utility across the critical HTN intervention threshold range (15–50% probability). DCA (S2 Fig) demonstrated superior net benefit for the “+METS-IR” model (green curve) versus the basic model (red curve) in this range, with maximal advantage observed at intermediate risk thresholds. This confirms METS-IR’s value for optimizing preventive strategies despite modest discrimination gains.

**Table 3:** Enhancement of Hypertension Discrimination and Risk Reclassification Through the Incorporation of METS-IR.

	C-statistic (95% CI)	*P*-value	NRI (95% CI)	*P*-value	IDI (95% CI)	*P*-value
Basic model	0.676 (0.662, 0.689)	< 0.001	Ref		Ref	
Basic model + METS-IR	0.680 (0.666, 0.693)	< 0.001	0.1658 (0.1033, 0.2283)	<0.001	0.0075 (0.0047, 0.0103)	<0.001

Basic model include age, gender, SBP. IDI: integrated discrimination improvement, NRI: net reclassification improvement, CI: confidence interval, Ref: reference, METS-IR: metabolic score for insulin resistance.

### Sensitivity analysis

Three sensitivity analyses were performed to evaluate the robustness of our findings. After excluding participants with a BMI ≥ 24 kg/m², the risk of HTN was 1.23 times higher than in the Q1 group (95% CI: 1.02–1.48). After excluding participants with pre-existing diabetes at baseline, the risk of HTN in the Q4 group was 1.62 times higher than in the Q1 group (95% CI: 1.39–1.88). After excluding participants with diabetes and overweight, the risk of HTN in the Q4 group was 1.25 times higher than in the Q1 group (95% CI 1.03–1.51). Table 4 demonstrates that the relationship between baseline METS-IR and the risk of HTN remained consistent even after excluding participants who were either overweight, DM, or both. Across all sensitivity cohorts, Schoenfeld tests indicated no PH violation (global *P* > 0.05; METS-IR-specific *P* > 0.05).

**Table 4:** Association between METS-IR and hypertension in different sensitivity analyses.

	Excluded participants with BMI ≥ 24 kg/m² (n = 2668)		Excluded participants with diabetes (n = 3899)		Excluded participants with BMI ≥ 24 kg/m² or diabetes (n = 2590)	
	HR (95% CI)	*P*-value	HR (95% CI)	*P*-value	HR (95% CI)	*P*-value
Person-years/events	19897/935		28421/1496		19353/897	
Per SD increase	1.10 (1.03, 1.17)	0.003	1.21 (1.15, 1.27)	<0.001	1.10 (1.03, 1.17)	0.005
METS-IR quartile						
Q1	Ref		Ref		Ref	
Q2	1.08 (0.89, 1.30)	0.452	1.09 (0.94, 1.27)	0.268	1.11 (0.92, 1.35)	0.270
Q3	1.23 (1.02, 1.48)	0.032	1.32 (1.14, 1.54)	<0.001	1.26 (1.05, 1.53)	0.015
Q4	1.23 (1.02, 1.48)	0.032	1.62 (1.39, 1.88)	<0.001	1.25 (1.03, 1.51)	0.023

HR: hazard ratios, CI: confidence interval, Ref: reference, METS-IR: metabolic score for insulin resistance. Model was adjusted for age, gender, marital status, rural residence, smoking status and drinking status, BUN, serum creatinine, TC, LDL-C, CRP, UA, dyslipidemia, heart disease.

### Subgroup analysis

**Fig 4:**
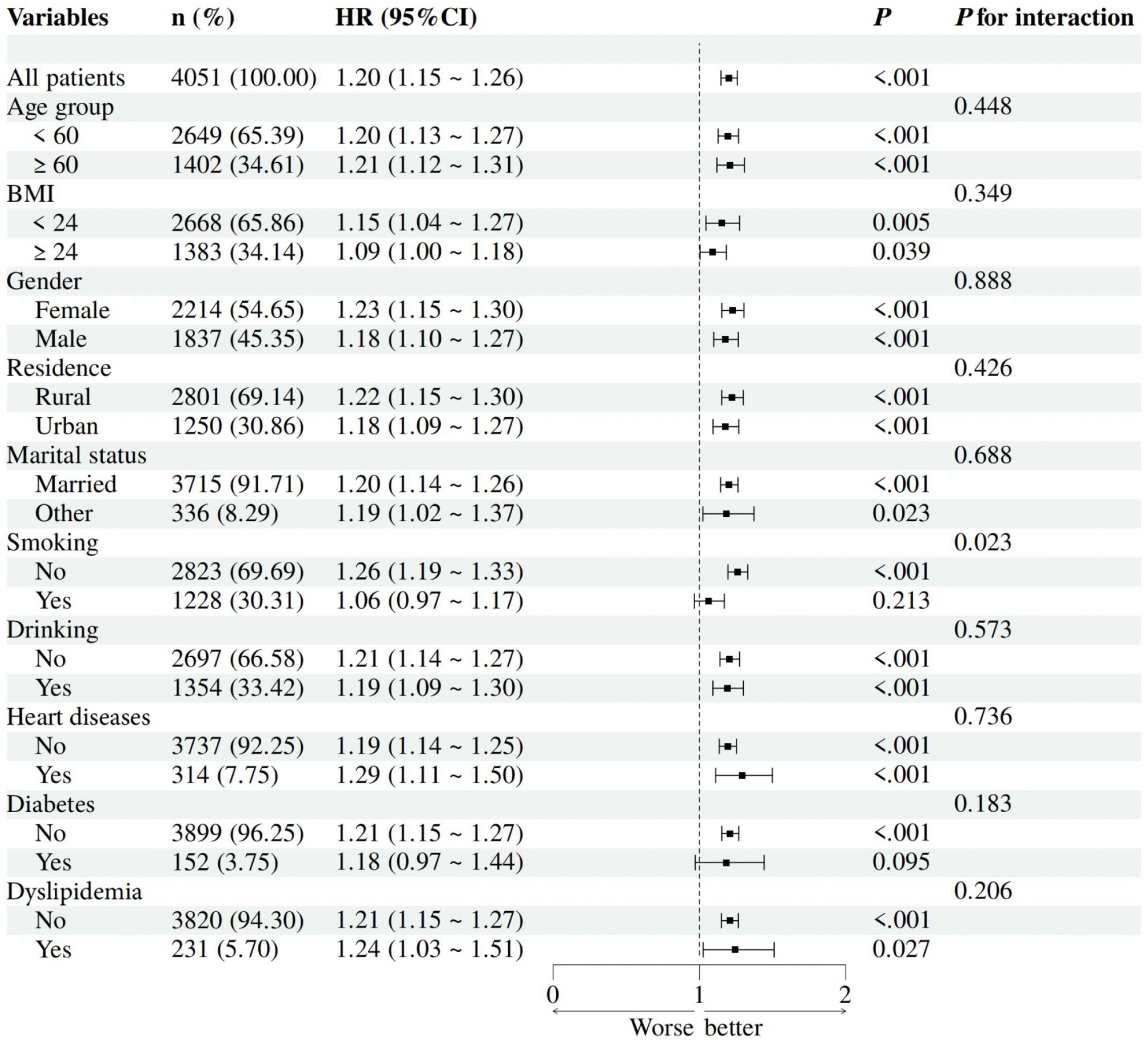
Subgroup and interaction analyses between the METS-IR and HTN across various subgroups. Multivariable cox regression analyses were performed across various populations, with adjustments made for age, gender, marital status, rural residence, smoking status and drinking status, BUN, serum creatinine, TC, LDL-C, CRP, UA, dyslipidemia, heart disease, diabetes mellitus.

To further explore the relationship between baseline METS-IR and the initial occurrence of HTN, we performed subgroup analyses stratified by potential risk factors. **Fig 4** illustrates consistent associations between higher METS-IR levels and an increased prevalence of HTN across various subgroups (after adjusting for multiple covariates), including age, sex, BMI, marital status, region, alcohol consumption, dyslipidemia, DM, and heart disease (interaction *P*-values > 0.05). METS-IR significantly predicted HTN in all subgroups (*P* < 0.05) except DM (HR = 1.18, 95% CI: 0.97–1.44; *P* = 0.095). Smoking status modified this relationship (interaction *P* = 0.023), with stronger effects in non-smokers (HR = 1.26, 95% CI: 1.19–1.33) versus smokers (HR = 1.06, 95% CI: 0.97–1.17). No interactions were detected for age, BMI, gender, or other factors (all *P* > 0.05).

### Stratified analysis

As detailed in S5 Table, the positive association between METS-IR and the risk of incident HTN was consistent in both males and females when analyzed separately. In the fully adjusted model (model 3), the hazard ratio for each 1-SD increase in METS-IR remained significant for both males (HR = 1.09, 95% CI: 1.01–1.17) and females (HR = 1.16, 95% CI: 1.08–1.24). A significant dose-response trend was observed in females (*P* for trend < 0.001), with participants in the highest METS-IR quartile (Q4) exhibiting a 43% increased risk of HTN (HR = 1.43, 95% CI: 1.16–1.76) compared to the lowest quartile (Q1). For males, the trend was positive but not statistically significant in model 3 (*P* for trend = 0.085). This stratified analysis suggests a consistent pattern of association between METS-IR and HTN risk in both genders, with a statistically significant effect observed in females and a positive trend noted in males, collectively supporting the main findings of our study.

When stratified by smoking status, the association between METS-IR and HTN risk remained robust and significant in non-smokers (HR = 1.19 per 1-SD increase, 95% CI: 1.12–1.26, *P* < 0.001). However, this association was attenuated and non-significant among smokers (HR = 1.00, 95% CI: 0.91–1.10, *P* = 0.966), suggesting that smoking status may be an important effect modifier (*P*-for-interaction = 0.023) as indicated in our initial subgroup analysis. Furthermore, stratification by SBP (<120 vs. ≥ 120 mmHg) [30] demonstrated that the positive association between METS-IR and HTN risk was consistent and significant across both strata. These findings confirm the stability of the METS-IR effect concerning SBP and delineate its particular relevance in non-smoking populations. Detailed results are available in S6 Table and S7 Table.

## Discussion

This nationwide prospective cohort study of 4,051 baseline normotensive Chinese adults (≥45 years) demonstrated that elevated baseline METS-IR—a non-insulin metric integrating BMI, TG, HDL-C, and FBG—independently predicted incident HTN over 9 years. After full adjustment for confounders (including demographics, lifestyle factors, comorbidities, biomarkers, and BP in Model 3), participants in the highest METS-IR quartile (Q4) had a 31% higher HTN risk versus the lowest quartile (Q1: HR = 1.31, 95% CI: 1.13–1.52; *P* for trend < 0.001). A linear dose-response relationship was confirmed (*P* for overall trend < 0.001; *P* for nonlinear = 0.310). Adding METS-IR to conventional predictors (age/sex/SBP) improved HTN prediction (NRI = 16.58%, IDI = 0.75%; both *P* < 0.001). Results remained robust after excluding overweight individuals or DM. METS-IR offers a practical risk-stratification tool in resource-limited settings, requiring only routine metabolic measures. Lower METS-IR levels are associated with reduced HTN incidence, supporting its utility for early risk identification and preventive interventions in China—where HTN affects ~244 million adults, yet control rates remain critically low (15.3%)—to mitigate CVD burden at the population level [2].

IR is a key pathophysiological mechanism in HTN development [31]. The METS-IR—a non-insulin index integrating BMI, TG, HDL-C, and FBG—accurately reflects metabolic dysregulation without requiring fasting insulin measurements, enhancing clinical feasibility. METS-IR demonstrates significant associations with pre-diabetes [32], DM [33], CVD [34], stroke [35], and hyperuricemia [10], outperforming other non-insulin-based IR surrogates in differential diagnosis [8]. Cross-sectional NHANES data link METS-IR to HTN prevalence in U.S. adults [36], aligning with longitudinal evidence. A Chinese cohort study confirmed elevated METS-IR predicts HTN risk in non-overweight adults [37]. This study’s sensitivity analysis revealed this association persists in normal-BMI individuals, potentially due to reduced subcutaneous fat promoting ectopic lipid deposition and hypertriglyceridemia [38]. The lower prevalence of traditional risk factors in higher METS-IR quartiles may arise from selection bias, differential health-seeking behaviors, and metabolic susceptibility, supported by higher dyslipidemia/DM rates and persistent associations after exclusions. Notably, METS-IR correlates with HTN in high-stress populations like Thai police officers, highlighting its relevance in contexts with elevated CVD risk.

METS-IR demonstrates gender-dependent predictive value for cardiometabolic diseases, with studies indicating stronger associations in women [10,20]. This disparity may arise from sex hormone and adipokine variations enhancing insulin sensitivity [39–41]. Estrogen confers protection against IR [41], while polycystic ovary syndrome-related hormonal imbalances exacerbate dysregulation [42]. Although menopause-related estrogen decline may attenuate these effects, the current cohort (including postmenopausal women) showed no significant gender differences [20]. METS-IR also outperforms TG/HDL-C and TyG indices in prehypertension prediction, particularly in males [8], and correlates with incident isolated diastolic hypertension (IDH)—a key CVD risk factor prevalent in young/middle-aged Asians [43,44].

East Asian meta-analyses confirm a dose-response relationship between METS-IR and HTN risk [45]. Bidirectional HTN-IR links are suggested by HOMA-IR’s 43% elevated HTN risk [46], though compensatory hyperinsulinemia may initially mitigate metabolic disruption [37]. Mendelian randomization studies provide causal evidence: each 1-SD increase in genetically predicted IR elevates hypertension risk by 6% [47].

Smoking exacerbates IR by elevating insulin/blood glucose levels [48], independently increasing HTN risk even after adjusting for classical confounders [49]. Crucially, smoking interacts synergistically with occupational stress (e.g., amplified HTN risk in Sinopec workers [50]) and other lifestyle factors (alcohol/BMI), necessitating integrated prevention strategies targeting smoking cessation and stress mitigation.

All four sub-indicators of the METS-IR-BMI, HDL-C, TG, and FBG—contribute significantly to the pathogenesis of HTN, potentially through mechanisms involving the IR pathway. Elevated BMI significantly contributes to HTN pathogenesis through IR pathways, correlating strongly with increased BP and CVD risk. A longitudinal study of 1,283 children demonstrated that higher BMI at ages 9−11 predicts elevated systolic and diastolic BP at age 11 [51]. A meta-analysis of 419,488 individuals confirmed that HTN’s cardiovascular risk escalates across all BMI subgroups [52]. Clinically, elevated BMI associates with refractory HTN, suggesting obesity-linked treatment resistance [53], a finding reinforced by Iran’s nationwide study where obese hypertensive patients (BMI ≥ 30) showed 69.7% uncontrolled HTN prevalence versus 28.1% in normal-weight counterparts [54]. Mechanistically, high-fat diets induce angiotensin-converting enzyme 1 (ACE1) overexpression in hypertensive rats, directly linking adiposity to BP dysregulation [55]. IR mediates obesity-related comorbidities including end-stage kidney disease, acting synergistically with HTN and hyperuricemia [56]. Adipocytokines (leptin, IL-6) and pro-inflammatory cytokines further amplify IR and HTN in T2DM patients [57], establishing insulin sensitivity management as vital for HTN control.

TG and HDL-C collectively influence HTN via dyslipidemia and IR. Elevated TG—a hallmark of IR—promotes dyslipidemia and CVD risk through impaired sodium excretion and vascular dysfunction [58]. TG exacerbates IR, creating a pathological cycle that elevates BP, with pro-inflammatory cytokines further disrupting TG metabolism and insulin signaling [59]. Hypertensive individuals exhibit 30% higher fasting TG and significantly delayed postprandial clearance versus normotensive controls [60]. Conversely, reduced HDL-C—prevalent in HTN and T2DM—diminishes nitric oxide release and increases monocyte-endothelial adhesion, elevating vascular resistance [61,62]. Critically, insulin therapy in T2DM improves HDL-C quantity but fails to restore functional reverse cholesterol transport [63]. Mendelian randomization studies confirm the TG/HDL-C ratio (reflecting IR) causally contributes to diabetes and HTN development, with identified genetic loci enabling deeper mechanistic exploration [63].

FBG dysregulation directly fuels HTN via IR-mediated pathways. Diminished insulin response elevates blood glucose, driving BP increases. In hypertensive T2DM patients, heightened IR correlates with elevated BP and pro-inflammatory cytokines [64]. Pediatric studies confirm this association: among obese children, IR and glucose alterations directly link to HTN and Renin-angiotensin-aldosterone system (RAAS) activation [65]. These findings underscore the necessity for early interventions targeting insulin sensitivity and glycemic control to mitigate HTN risk across age groups. The METS-IR framework integrates these pathways into a unified risk stratification tool.

Overall, the metabolic repercussions of IR can potentially contribute to the onset of HTN via multiple pathways. The excessive stimulation of the carotid body induced by IR, accompanied by compensatory hyperinsulinemia, results in the activation of the sympathetic nervous system and the subsequent release of adrenaline and noradrenaline. In this context, the activation of the sympathetic nervous system not only impacts cardiovascular function but may also exacerbate metabolic abnormalities by promoting endothelial dysfunction and augmenting BP responses [66]. Furthermore, IR activates the RAAS, thereby promoting sodium reabsorption in the renal tubules. This process subsequently leads to plasma volume expansion and elevated BP [67]. Simultaneously, impaired insulin signaling pathways can result in endothelial dysfunction and a reduction in nitric oxide synthase activity, subsequently resulting in systemic vasoconstriction [68].

Our study offers several notable advantages. First, the research presents a prospective investigation to elucidate the association between METS-IR and the incidence of HTN among Chinese population. The comprehensive data set and extended follow-up period in this analysis enhance the reliability and generalizability of the findings. Second, in contrast to cross-sectional studies, this longitudinal investigation of middle-aged and elderly populations provides a more robust validation of the relationship between METS-IR and the prevalence of HTN, thereby supporting a prospective association consistent with a causal relationship.

Several limitations are inherent to the current study. First, while some potential confounders were adjusted for, biases from unmeasured confounders persist, including dietary sodium/potassium intake, physical activity patterns, medication use (e.g., NSAIDs, steroids), sleep apnea severity, genetic factors, and psychosocial stressors, remain unaccounted for. Second, since insulin markers were not directly measured in the study population, we were unable to assess the predictive capability of METS-IR against markers that directly reflect IR. Third, the anonymization of detailed geographical information in the public CHARLS dataset limited our ability to account for the complex survey design (e.g., clustering and stratification) in the primary analyses. Therefore, the standard errors in our Cox models may be underestimated, although the HR estimates should remain valid. Forth, The data were derived from the Chinese population, and it is uncertain if there are ethnic variations in these associations. Fifth, the underlying mechanisms linking METS-IR to HTN remain inadequately understood, necessitating further in-depth exploration in future studies. Sixth, although IPCW analysis suggests robustness to attrition bias, residual confounding from unmeasured factors related to early dropout cannot be excluded; however, the comparability of METS-IR components between retained and excluded participants substantially mitigates concern for selection bias affecting our primary exposure. Seventh, despite testing multiple interactions, we did not apply statistical corrections for multiplicity. These findings regarding interactions, particularly the significant finding with smoking status, are exploratory and require validation in independent cohorts.

## Conclusions

This investigation uncovers a substantial positive association between METS-IR and the prevalence of HTN among Chinese population. HTN is not merely a primary risk factor for CVD but is also closely linked to metabolic disorders. Monitoring METS-IR levels could facilitate the early detection of HTN risk, thereby providing a scientific foundation for preventive and interventional strategies.

## Supporting information

S1 Fig Missing-Data Matrix by Variable Combinations. (TIF)

S2 Fig Decision curve analysis comparing net benefit of hypertension prediction models. (TIF)

S1 Table Results of Global and Variable-Specific Schoenfeld Tests. (DOCX)

S2 Table Hazard Ratios for METS-IR: Comparison of Multiple Imputation and Complete Case Approaches. (DOCX)

S3 Table Baseline Characteristics of Excluded Participants (Lost follow-up) and Retained Participants. (DOCX)

S4 Table IPCW-weighted Cox model for METS-IR and hypertension risk. (DOCX)

S5 Table Association Between METS-IR and Hypertension Stratified by Sex. (DOCX)

S6 Table Association Between METS-IR and Hypertension Stratified by Smoking. (DOCX)

S7 Table Association Between METS-IR and Hypertension Stratified by SBP (Cutoff: 120 mmHg). (DOCX)
