# Supplementary material for: Relationship of the metabolic score for insulin resistance and the new-onset hypertension: Evidence from CHARLS
Source: PLoS One. 2025 Nov 7;20(11):e0336388. doi: 10.1371/journal.pone.0336388 (PMC12594336; doi:10.1371/journal.pone.0336388)
Supplement: S1 Table — (DOCX) [file pone.0336388.s003.docx]

**S1 Table** Results of Global and Variable-Specific Schoenfeld Tests

| **Variables** | **Chi-Square** | ***P* value** |
| --- | --- | --- |
| METS-IR | 3.11 | 0.078 |
| Age | 2.70 | 0.100 |
| Gender | 3.89 | 0.049 |
| Residence | 1.77 | 0.183 |
| Marital status | 1.05 | 0.306 |
| Smoking | 5.03 | 0.025 |
| Drinking | 0.60 | 0.440 |
| Diabetes mellitus | 0.94 | 0.332 |
| Heart diseases | 2.69 | 0.101 |
| Dyslipidemia | 2.28 | 0.131 |
| CRP | 0.23 | 0.630 |
| HbA1c | 0.06 | 0.805 |
| TC | 0.78 | 0.379 |
| LDL-C | 0.32 | 0.569 |
| BUN | 0.45 | 0.502 |
| Creatinine | 0.27 | 0.606 |
| UA | 2.16 | 0.142 |
| DBP | 2.60 | 0.107 |
| SBP | 5.28 | 0.022 |
| GLOBAL | 27.03 | 0.104 |
