# Supplementary material for: Relationship of the metabolic score for insulin resistance and the new-onset hypertension: Evidence from CHARLS
Source: PLoS One. 2025 Nov 7;20(11):e0336388. doi: 10.1371/journal.pone.0336388 (PMC12594336; doi:10.1371/journal.pone.0336388)
Supplement: S2 Table — (DOCX) [file pone.0336388.s004.docx]

**S2 Table** Hazard Ratios for METS-IR: Comparison of Multiple Imputation and Complete Case Approaches

|  | HR | 95% CI | *P* value |
| --- | --- | --- | --- |
| Multiple Imputation (m=20) | 1.19 | 1.15, 1.24 | <0.001 |
| Complete Case Analysis | 1.21 | 1.15, 1.27 | <0.001 |

HR: hazard ratios, CI: confidence interval, Ref: reference, METS-IR: metabolic score for insulin resistance.

Model adjusted for age, gender, marital status, rural residence, smoking status and drinking status, BUN, serum creatinine, TC, LDL-C, CRP, UA, dyslipidemia, heart disease and diabetes mellitus.
