# Supplementary material for: Relationship of the metabolic score for insulin resistance and the new-onset hypertension: Evidence from CHARLS
Source: PLoS One. 2025 Nov 7;20(11):e0336388. doi: 10.1371/journal.pone.0336388 (PMC12594336; doi:10.1371/journal.pone.0336388)
Supplement: S3 Table — (DOCX) [file pone.0336388.s005.docx]

**S3 Table Baseline Characteristics of Excluded Participants (Lost follow-up) and Retained Participants**

| Variables | Overall  (n = 5451) | **Retained Participants**  (n = 4051) | **Excluded Participants**  (n = 1400) | *P* value |
| --- | --- | --- | --- | --- |
| Age, years | 57.00 (50.00-63.00) | 56.00 (50.00-62.00) | 58.00 (51.00-66.00) | < 0.001 |
| Male, n (%) | 2591 (47.53%) | 1837 (45.35%) | 754 (53.86%) | < 0.001 |
| Rural residence, n (%) | 3641 (66.80%) | 2801 (69.14%) | 840 (60.00%) | < 0.001 |
| BMI (kg/m^2^) | 22.48 (20.40-24.83) | 22.61 (20.54-24.90) | 22.21 (20.07-24.63) | <0.001 |
| SBP, mmHg | 117.50 (109.00-126.50) | 117.00 (108.50-126.50) | 118.00 (109.50-127.50) | 0.018 |
| DBP, mmHg | 70.00 (64.00-76.50) | 70.00 (64.00-76.50) | 70.00 (64.00-76.50) | 0.748 |
| Married or living with spouse, n (%) | 4917 (90.20%) | 3715 (91.71%) | 1202 (85.86%) | <0.001 |
| Drinking, n (%) | 1865 (34.21%) | 1354 (33.42%) | 511 (36.50%) | 0.036 |
| Smoking, n (%) | 1737 (31.87%) | 1228 (30.31%) | 509 (36.36%) | < 0.001 |
| BUN, mg/dL | 15.15 (12.53-18.14) | 15.10 (12.52-18.12) | 15.27 (12.58-18.15) | 0.271 |
| FBG, mg/dL | 100.98 (93.42-110.52) | 100.98 (93.60-110.16) | 101.43 (93.06-112.14) | 0.289 |
| Creatinine, mg/dL | 0.75 (0.64-0.87) | 0.75 (0.64-0.87) | 0.76 (0.66-0.89) | <0.001 |
| TC, mg/dL | 187.89 (165.08-213.02) | 188.66 (165.85-213.02) | 185.57 (163.15-212.24) | 0.064 |
| TG, mg/dL | 98.24 (71.68-141.60) | 98.24 (71.68-141.60) | 100.00 (72.57-143.37) | 0.473 |
| HDL-C, mg/dL | 51.03 (41.37-61.08) | 51.03 (41.75-61.08) | 50.26 (41.37-61.86) | 0.778 |
| LDL-C, mg/dL | 113.27 (92.40-134.92) | 113.66 (93.17-135.70) | 110.57 (90.75-132.60) | 0.008 |
| CRP, mg/L | 0.90 (0.50-1.90) | 0.87 (0.49-1.77) | 1.00 (0.53-2.33) | <0.001 |
| HbA1c, % | 5.10 (4.90-5.40) | 5.10 (4.90-5.40) | 5.10 (4.90-5.40) | 0.787 |
| UA, mg/dL | 4.19 (3.50-5.00) | 4.14 (3.46-4.94) | 4.35 (3.62-5.18) | <0.001 |
| Diabetes mellitus, n (%) | 215 (3.94%) | 152 (3.75%) | 63 (4.50%) | 0.215 |
| Heart diseases, n (%) | 455 (8.35%) | 314 (7.75%) | 141 (10.07%) | 0.007 |
| Dyslipidemia, n (%) | 311 (5.71%) | 231 (5.70%) | 80 (5.71%) | 0.987 |
| METS-IR | 32.82 (28.83-38.05) | 32.95 (29.03-38.15) | 32.49 (28.30-37.9) | 0.007 |

Data are means ± SD, median (interquartile range), or n (%).

Abbreviations: METS-IR: metabolic score for insulin resistance, BMI: body mass index, BUN: blood urea nitrogen, SBP: systolic blood pressure, DBP: diastolic blood pressure, FBG: fasting blood glucose, HDL-C: high density lipoprotein cholesterol, LDL-C: low density lipoprotein cholesterol, TC: total cholesterol, TG triglycerides, CRP: C-reactive protein, HbA1c: glycosylated hemoglobin A1c, UA: uric acid.
