# Supplementary material for: Relationship of the metabolic score for insulin resistance and the new-onset hypertension: Evidence from CHARLS
Source: PLoS One. 2025 Nov 7;20(11):e0336388. doi: 10.1371/journal.pone.0336388 (PMC12594336; doi:10.1371/journal.pone.0336388)
Supplement: S4 Table — (DOCX) [file pone.0336388.s006.docx]

**S4 Table IPCW-weighted Cox model for METS-IR and hypertension risk**

|  | Non-adjusted model | | Model 1 | | Model 2 | | Model 3 | |
| --- | --- | --- | --- | --- | --- | --- | --- | --- |
|  | HR (95% CI) | *P* value | HR (95% CI) | *P* value | HR (95% CI) | *P* value | HR (95% CI) | *P* value |
| Continues | | | | | | | | |
| Per SD increase | 1.17 (1.11, 1.22) | <0.001 | 1.20 (1.14, 1.25) | <0.001 | 1.16 (1.10, 1.23) | <0.001 | 1.08 (1.02, 1.15) | 0.008 |
| METS-IR quartile | | | | | | | | |
| Q1 | Ref |  | Ref |  | Ref |  | Ref |  |
| Q2 | 1.19 (1.02, 1.38) | 0.024 | 1.22 (1.05, 1.43) | 0.009 | 1.20 (1.03, 1.40) | 0.019 | 1.15 (0.99, 1.34) | 0.076 |
| Q3 | 1.33 (1.15, 1.54) | <0.001 | 1.42 (1.22, 1.64) | <0.001 | 1.35 (1.16, 1.57) | <0.001 | 1.18 (1.01, 1.38) | 0.032 |
| Q4 | 1.54 (1.33, 1.77) | <0.001 | 1.66 (1.43, 1.92) | <0.001 | 1.49 (1.27, 1.73) | <0.001 | 1.25 (1.06, 1.46) | 0.006 |
| *P* for trend |  | <0.001 |  | <0.001 |  | <0.001 |  | <0.001 |

HR: hazard ratios, CI: confidence interval, Ref: reference, METS-IR: metabolic score for insulin resistance.

Non-adjusted model adjusted for none.

Model 1 adjusted for age, gender, marital status, rural residence, smoking status and drinking status.

Model 2 adjusted for BUN, serum creatinine, TC, LDL-C, CRP, UA, dyslipidemia, heart disease and diabetes mellitus on the basis of Model 1.

Model 3 adjusted for SBP and DBP on the basis of Model 2.
