# Supplementary material for: Relationship of the metabolic score for insulin resistance and the new-onset hypertension: Evidence from CHARLS
Source: PLoS One. 2025 Nov 7;20(11):e0336388. doi: 10.1371/journal.pone.0336388 (PMC12594336; doi:10.1371/journal.pone.0336388)
Supplement: S5 Table — (DOCX) [file pone.0336388.s007.docx]

**S5 Table** Association Between METS-IR and Hypertension Stratified by Sex

| Sex | METS-IR | Non-adjusted model |  | Model 1 |  | Model 2 |  | Model 3 | |
| --- | --- | --- | --- | --- | --- | --- | --- | --- | --- |
|  |  | HR (95% CI) | *P* value | HR (95% CI) | *P* value | HR (95% CI) | *P* value | HR (95% CI) | *P* value |
| Male | Per SD increase | 1.16 (1.09, 1.24) | <0.001 | 1.20 (1.13, 1.28) | <0.001 | 1.17 (1.09, 1.25) | <0.001 | 1.09 (1.01, 1.17) | 0.021 |
|  | Quartile 1 | Ref |  | Ref |  | Ref |  | Ref |  |
|  | Quartile 2 | 0.86 (0.69, 1.07) | 0.183 | 0.91 (0.73, 1.13) | 0.388 | 0.91 (0.73, 1.14) | 0.424 | 0.86 (0.69, 1.08) | 0.198 |
|  | Quartile 3 | 1.13 (0.92, 1.39) | 0.244 | 1.24 (1.00, 1.52) | 0.046 | 1.20 (0.97, 1.49) | 0.086 | 1.15 (0.93, 1.43) | 0.192 |
|  | Quartile 4 | 1.34 (1.09, 1.63) | 0.005 | 1.50 (1.22, 1.85) | <0.001 | 1.39 (1.13, 1.72) | 0.002 | 1.11 (0.90, 1.38) | 0.327 |
|  | *P* for trend |  | <0.001 |  |  |  | <0.001 |  | 0.0848 |
| Female | Per SD increase | 1.19 (1.12, 1.26) | <0.001 | 1.24 (1.17, 1.31) | <0.001 | 1.23 (1.15, 1.31) | <0.001 | 1.16 (1.08, 1.24) | <0.001 |
|  | Quartile 1 | Ref |  | Ref |  | Ref |  | Ref |  |
|  | Quartile 2 | 1.09 (0.89, 1.34) | 0.423 | 1.16 (0.95, 1.43) | 0.146 | 1.15 (0.94, 1.42) | 0.182 | 1.07 (0.87, 1.32) | 0.519 |
|  | Quartile 3 | 1.21 (0.99, 1.48) | 0.058 | 1.37 (1.12, 1.68) | 0.002 | 1.34 (1.09, 1.65) | 0.006 | 1.20 (0.98, 1.48) | 0.080 |
|  | Quartile 4 | 1.64 (1.35, 1.98) | <0.001 | 1.92 (1.58, 2.33) | <0.001 | 1.80 (1.46, 2.21) | <0.001 | 1.43 (1.16, 1.76) | <0.001 |
|  | *P* for trend |  | <0.001 |  | <0.001 |  | <0.001 |  | <0.001 |

HR: hazard ratios, CI: confidence interval, Ref: reference, METS-IR: metabolic score for insulin resistance.

Non-adjusted model adjusted for none.

Model 1 adjusted for age, marital status, rural residence, smoking status and drinking status.

Model 2 adjusted for BUN, serum creatinine, TC, LDL-C, CRP, UA, dyslipidemia, heart disease and diabetes mellitus on the basis of Model 1.

Model 3 adjusted for SBP and DBP on the basis of Model 2.
