# Supplementary material for: Relationship of the metabolic score for insulin resistance and the new-onset hypertension: Evidence from CHARLS
Source: PLoS One. 2025 Nov 7;20(11):e0336388. doi: 10.1371/journal.pone.0336388 (PMC12594336; doi:10.1371/journal.pone.0336388)
Supplement: S6 Table — (DOCX) [file pone.0336388.s008.docx]

| Smoking | METS-IR | Non-adjusted model |  | Model 1 |  | Model 2 |  | Model 3 | |
| --- | --- | --- | --- | --- | --- | --- | --- | --- | --- |
|  |  | HR (95% CI) | *P* value | HR (95% CI) | *P* value | HR (95% CI) | *P* value | HR (95% CI) | *P* value |
| Yes | Per SD increase | 1.08 (0.99, 1.17) | 0.079 | 1.12 (1.03, 1.21) | 0.011 | 1.06 (0.96, 1.16) | 0.253 | 1.00 (0.91, 1.10) | 0.966 |
|  | Quartile1 | Ref |  | Ref |  | Ref |  | Ref |  |
|  | Quartile2 | 0.79 (0.61, 1.02) | 0.074 | 0.83 (0.64, 1.08) | 0.161 | 0.82 (0.63, 1.07) | 0.145 | 0.79 (0.61, 1.03) | 0.085 |
|  | Quartile3 | 0.90 (0.70, 1.16) | 0.408 | 0.97 (0.76, 1.26) | 0.843 | 0.93 (0.72, 1.20) | 0.573 | 0.90 (0.69, 1.17) | 0.442 |
|  | Quartile4 | 1.09 (0.85, 1.39) | 0.491 | 1.21 (0.95, 1.56) | 0.129 | 1.03 (0.79, 1.34) | 0.828 | 0.86 (0.66, 1.13) | 0.279 |
|  | *P* for trend |  | 0.323 |  | 0.075 |  | 0.656 |  | 0.455 |
| No | Per SD increase | 1.22 (1.16, 1.28) | <0.001 | 1.27 (1.21, 1.34) | <0.001 | 1.26 (1.20, 1.33) | <0.001 | 1.19 (1.12, 1.26) | <0.001 |
|  | Quartile1 | Ref |  | Ref |  | Ref |  | Ref |  |
|  | Quartile2 | 1.13 (0.94, 1.36) | 0.192 | 1.21 (1.00, 1.45) | 0.0450 | 1.20 (0.99, 1.44) | 0.058 | 1.12 (0.93, 1.35) | 0.235 |
|  | Quartile3 | 1.36 (1.14, 1.62) | <0.001 | 1.54 (1.29, 1.85) | <0.001 | 1.52 (1.27, 1.82) | <0.001 | 1.39 (1.16, 1.67) | <0.001 |
|  | Quartile4 | 1.77 (1.50, 2.10) | <0.001 | 2.08 (1.75, 2.47) | <0.001 | 1.97 (1.65, 2.36) | <0.001 | 1.55 (1.29, 1.86) | <0.001 |
|  | *P* for trend |  | <0.001 |  | <0.001 |  | <0.001 |  | <0.001 |

**S6 Table** Association Between METS-IR and Hypertension Stratified by Smoking

HR: hazard ratios, CI: confidence interval, Ref: reference, METS-IR: metabolic score for insulin resistance.

Non-adjusted model adjusted for none.

Model 1 adjusted for age, gender, marital status, rural residence and drinking status.

Model 2 adjusted for BUN, serum creatinine, TC, LDL, CRP, UA, dyslipidemia, heart disease and diabetes mellitus on the basis of Model 1.

Model 3 adjusted for SBP and DBP on the basis of Model 2.
