# Supplementary material for: Relationship of the metabolic score for insulin resistance and the new-onset hypertension: Evidence from CHARLS
Source: PLoS One. 2025 Nov 7;20(11):e0336388. doi: 10.1371/journal.pone.0336388 (PMC12594336; doi:10.1371/journal.pone.0336388)
Supplement: S7 Table — (DOCX) [file pone.0336388.s009.docx]

| SBP | METS-IR | Non-adjusted model |  | Model 1 |  | Model 2 |  | Model 3 | |
| --- | --- | --- | --- | --- | --- | --- | --- | --- | --- |
|  |  | HR (95% CI) | *P* value | HR (95% CI) | *P* value | HR (95% CI) | *P* value | HR (95% CI) | *P* value |
| ≥120 mmHg | Per SD increase | 1.12 (1.05, 1.19) | <0.001 | 1.17 (1.09, 1.24) | <0.001 | 1.16 (1.08, 1.24) | <0.001 | 1.15 (1.07, 1.23) | <0.001 |
|  | Quartile1 | Ref |  | Ref |  | Ref |  | Ref |  |
|  | Quartile2 | 1.01 (0.84, 1.22) | 0.912 | 1.07 (0.89, 1.30) | 0.456 | 1.07 (0.88, 1.30) | 0.485 | 1.07 (0.89, 1.30) | 0.471 |
|  | Quartile3 | 1.29 (1.08, 1.55) | 0.005 | 1.44 (1.20, 1.74) | <0.001 | 1.41 (1.17, 1.71) | <0.001 | 1.41 (1.16, 1.70) | <0.001 |
|  | Quartile4 | 1.22 (1.02, 1.46) | 0.034 | 1.38 (1.14, 1.68) | <0.001 | 1.33 (1.09, 1.62) | 0.005 | 1.30 (1.07, 1.59) | 0.009 |
|  | *P* for trend |  | 0.004 |  | <0.001 |  | <0.001 |  | 0.001 |
| < 120 mmHg | Per SD increase | 1.14 (1.06, 1.22) | <0.001 | 1.19 (1.12, 1.27) | <0.001 | 1.16 (1.08, 1.24) | <0.001 | 1.13 (1.05, 1.21) | 0.001 |
|  | Quartile1 | Ref |  | Ref |  | Ref |  | Ref |  |
|  | Quartile2 | 0.89 (0.70, 1.12) | 0.301 | 0.95 (0.75, 1.20) | 0.672 | 0.95 (0.75, 1.19) | 0.643 | 0.92 (0.73, 1.16) | 0.479 |
|  | Quartile3 | 0.93 (0.74, 1.17) | 0.559 | 1.05 (0.84, 1.33) | 0.665 | 1.03 (0.81, 1.30) | 0.811 | 0.97 (0.77, 1.23) | 0.804 |
|  | Quartile4 | 1.32 (1.06, 1.63) | 0.011 | 1.56 (1.25, 1.93) | <0.001 | 1.41 (1.12, 1.76) | 0.003 | 1.29 (1.03, 1.62) | 0.028 |
|  | *P* for trend |  | 0.008 |  | <0.001 |  | 0.002 |  | 0.022 |

**S7 Table** Association Between METS-IR and Hypertension Stratified by SBP (Cutoff: 120 mmHg)

HR: hazard ratios, CI: confidence interval, Ref: reference, METS-IR: metabolic score for insulin resistance.

Non-adjusted model adjusted for none.

Model 1 adjusted for age, gender, marital status, rural residence, smoking status and drinking status.

Model 2 adjusted for BUN, serum creatinine, TC, LDL, CRP, UA, dyslipidemia, heart disease and diabetes mellitus on the basis of Model 1.

Model 3 adjusted for DBP on the basis of Model 2.
